# Narciclasine attenuates LPS-induced acute lung injury in neonatal rats through suppressing inflammation and oxidative stress

**DOI:** 10.1080/21655979.2020.1795424

**Published:** 2020-07-21

**Authors:** Qingning Duan, Yin Jia, Yan Qin, Yingji Jin, Haozhong Hu, Jiebin Chen

**Affiliations:** Department of Pediatrics, Hospital Affiliated 5 to Nantong University (Taizhou People’s Hospital), Taizhou, Jiangsu, PR China

**Keywords:** Narciclasine, inflammation, oxidative stress, acute lung injury, TLR4/NF-κB/Cox2

## Abstract

Acute lung injury (ALI) is a life-threatening disorder related to serious pulmonary inflammation. Narciclasine exhibits strong anti-inflammation activity and attenuates the reactive oxygen species (ROS) production. The present study aims to investigate the underlying mechanism related to the effect of narciclasine on the pathogenesis of neonatal acute lung injury (ALI). Narciclasine attenuated LPS-induced pathological injury and pulmonary edema. In addition, narciclasine suppressed the secretion of inflammatory cytokines, including necrosis factor-α (TNF-α), Interleukin (IL-6), IL-1β, monocyte chemotactic protein-1 (MCP-1) in serum, and inhibited the expressions of intercellular adhesion molecule-1 (ICAM-1) and vascular cell adhesion molecule-1 (VCAM-1) in lung tissues of neonatal ALI rats. Furthermore, narciclasine alleviated oxidative stress and apoptosis in lung tissues. Importantly, narciclasine exerted an inhibition effect on NF-κB nuclear translocation and activation of Toll-like Receptor 4 (TLR4)/Nuclear factor (NF)-κB/Cyclooxygenase 2 (Cox2) signaling pathway. Taken together, narciclasine protected against lung injury via inhibition effect on excessive inflammation, oxidative stress and apoptosis, hence, narciclasine may be considered as an effective and novel agent for clinical therapeutic strategy of ALI Treatment.

## Graphical abstract

**Figure:**
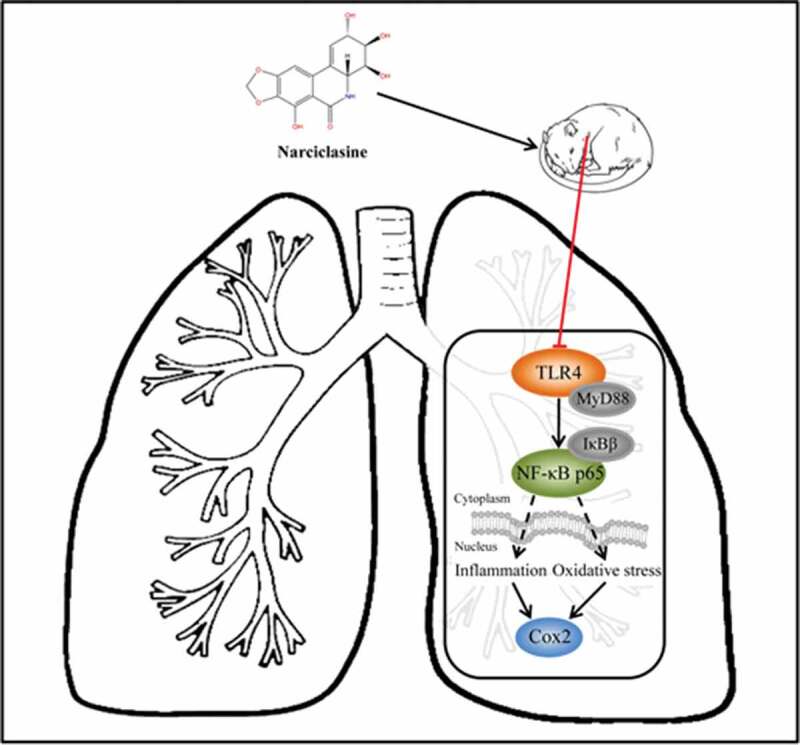

## Introduction

Acute lung injury (ALI) is a life-threatening disorder with high incidence and mortality, which is related to serious pulmonary inflammation [1]. ALI is characterized by increased permeability, excessive airway inflammatory response and impaired integrity of lung tissues structure, which causes alveolar congestion, hemorrhage, hypoxemia, edema and atelectasis [2]. Previous studies have reported that neonates are especially susceptible to pulmonary injury which results in high mortality rate in preterm infants [3–5]. It has been well established that uncontrolled pulmonary inflammatory response is closely related to pathogenesis of ALI on infants [6]. Great progress has been achieved in understanding the potential biochemical mechanism of ALI, while current clinical immune-targeted or pharmacologic therapies still exhibit numerous limitations on reducing mortality rate of ALI patients. Hence, it is urgent to develop novel and effectively therapeutic strategies for neonatal ALI.

Chinese traditional herbs have made a great contribution to therapeutically effective agents for numerous clinical disorders due to eminent biochemical activities [7,8]. Narciclasine, also known as lycoricidinol, is a natural compound of *Haemanthus coccineus* L belonging to the Amaryllidaceae family, which is known for its anticancer properties previously [9]. Recent studies have reported that narciclasine exhibits strong anti-inflammation activity *in vitro* and *in vivo* [10]. Narciclasine effectively suppressed inflammatory response through inhibiting the interaction of leukocytes with endothelial cells and inactivation of TNF-mediated signaling pathways [11]. In addition, earlier studies have demonstrated that narciclasine attenuates the reactive oxygen species (ROS) production in muscle tissue of diet-induced obesity mice [12]. However, the role of narciclasine in ALI progression remains not defined. Based on the above evidence, we hypothesize that narciclasine has a potential therapeutic effect on neonatal ALI.

In the present study, LPS-induced newborn rat models were established to investigate the molecular mechanism underlying the therapeutic effect of narciclasine on ALI. Our findings provide evidence to demonstreate the anti-inflammation action of narciclasine in pulmonary injury, which may be a novel therapeutic target in clinical therapeutic strategy of neonatal ALI.

## Materials and methods

### Animals and preparation of ALI model

All animal care and experimental procedures were conducted in accordance with the guidelines approved by the Animal Ethical Committee of Taizhou People’s Hospital. Sprague-Dawley rats (3–8 days old, 8–14 g bodyweight) were obtained from Oriental Bio Service Inc. (Nanjing). All rats were fed on standard chow diet *ad libitum* and housed in a room maintained at 22°C ± 2°C with 12 h light/dark cycle and 55%–65% relative humidity.

Neonatal rats were induced as previously described with minor revision [13]. Seventy-five neonatal rats were randomly allocated into three groups, including control group (*n* = 15), narciclasine group (*n* = 15), and LPS-induced ALI group (*n* = 45). The rats were intraperitoneally injected with equal volume of normal saline used as control group. Narciclasine (0.7 mg/kg, 200 μL), obtained from Carl Roth (Karlsruhe, Germany), was subcutaneously injected into the neck of rats belonging to narciclasine group [11]. Rats in the LPS-induced group were injected intraperitoneally with LPS (2 mg/kg) purchased from Sigma-Aldrich (St. Louis, Mo.). Subsequently, thirty infected rats were randomly divided into three groups with 15 animals each, including one LPS-induced ALP group, one LPS + vehicle (10% Kolliphor and 1% DMSO in 0.9% sodium chloride solution) group, and one LPS (2 mg/kg) + narciclasine (0.7 mg/kg) group. Rats were subjected to intraperitoneal injections of narciclasine or vehicle 12 h prior to LPS exposure, according to the previous study [11]. At the end of study, all rats were sacrificed 72 h after LPS or saline treatment and the blood samples were collected. Any animal reaching humane endpoints were euthanized using CO_2_ inhalation followed by cervical dislocation under the supervision of attending veterinarian.

### Hematoxylin-eosin (H&E) staining

The samples of the whole left lung were fixed in 4% paraformaldehyde for 24 h at room temperature. After being embedded in paraffin, lung tissues were sectioned into 5-µm-thick slices and stained with hematoxylin and eosin. Finally, the sections were observed and captured using an inverted light microscope (Leica Microsystems, Wetzlar, Germany) at the 200× magnification to analyze the morphological characteristics of lung tissues.

### Pulmonary edema score

Pulmonary edema score indicates the degree of pleural effusion after lung injury by calculating the lung wet/dry weight ratio [14]. The wet weight of the right lung was measured immediately after elimination of foreign matter. Subsequently, the lung tissues were dried in an oven at 80°C for 72 h or until tissues were completely dry and assessed immediately to calculate the dry weight.

### Cytokine measurement

After cotreatment of LPS and narciclasine, blood samples of each group were collected through eyeball extraction, and serum samples were obtained through centrifugation at 4000 rpm for 10 min at 4°C. The expressions of tumor necrosis factor α (TNF-α) interleukin 6 (IL-6), interleukin 1β (IL-1β), and monocyte chemotactic protein-1 (MCP-1) in serum were determined using ELISA assay kits (Invitrogen, Carlsbad, CA, USA) according to the manufacturer’s instructions. The color intensity was assayed at 450 nm using a microplate reader (Bio-Rad, Hercules, CA, USA).

### Western blot

Cytosolic and nuclear proteins were obtained using the cytoplasmic protein extraction agent and nuclear protein extraction agent, respectively (Beyotime Biotech, Guangzhou, China). Then total protein (25 μg) was separated by SDS-PAGE and transferred to PVDF membranes (Merck Millipore, Billerica, MA). After being blocked with 5% nonfat milk and washed by TBST three times, the membranes were incubated at 4 °C overnight with appropriate primary antibodies against ICAM-1 (dilution, 1:1,000), VCAM-1 (dilution, 1:1,000), Bax (dilution, 1:1,000), cleaved caspase3 (dilution, 1:500), cleaved caspase9 (dilution, 1:500), Bcl2 (dilution, 1:1,000), TLR4 (dilution, 1:1,000), MyD88 (dilution, 1:1,000), Cox2 (dilution, 1:1,000), NF-κb p65 (dilution, 1:1,000), p-IκBβ (dilution, 1:5,000), IκBβ (dilution, 1:1,000). All primary antibodies were purchased from Cell Signaling Technology (Danvers, MA, USA). Thereafter, PVDF membranes were incubated with the corresponding horseradish peroxidase-conjugated secondary antibody at room temperature for 2 h and visualized by an ECL Plus detection system. The optical densities of bands were presented as their ratios to control lanes, determined with ImageJ software.

### Analyzes for the oxidative stress status in lung tissue

Total antioxidant capacity of pulmonary tissues was analyzed. The amount of reactive oxygen species (ROS) and antioxidant enzymes activities, such as superoxide dismutase (SOD), lactate dehydrogenase (LDH), and myeloperoxidase (MPO) were detected using the specific commercial assay kits, according to the manufacturer’s recommendations. Briefly, ROS were quantified by Reactive oxygen species Assay Kit (cat.E004-1-1, Nanjing Jiancheng company). LDH activity was determined by using Lactate dehydrogenase Assay Kit (cat.A020-2-2, Nanjing Jiancheng company). MPO activity was measured by (cat.A044-1-1, Nanjing Jiancheng company). SOD activity was quantified with an SOD Assay Kit (cat.A001-3-2, Nanjing Jiancheng company). Subsequently, absorbance was measured by a microplate reader (Bio-Rad, Hercules, CA, USA).

### TUNEL assay

Cell apoptosis in lung tissues was detected using cell death assay kit (Roche, Mannheim, Germany). In brief, lung tissues were washed with PBS and fixed with 4% paraformaldehyde. After being blocked with 3% H_2_O_2_ for 15 min, fluorescein-isothiocyanate (FITC)-dUTP (green) and DAPI (4′, 6-diamidino-2-phenylindole) (blue) were used to stain the apoptotic cells and nuclei. The percentage of TUNEL positive cells to the total number of cells is defined as the cell apoptotic rate. A fluorescence microscope (Olympus Corp., Tokyo, Japan) was employed to capture the images (magnification, ×200).

### Statistical analysis

Statistical analysis was performed with GraphPad Prism 5.0 (La Jolla, CA, USA). All data were expressed as mean ± standard deviation (SD). Statistical differences between the groups were measured by one-way analysis of variance (ANOVA) followed by post hoc Bonferroni test. All experiments were repeated at least 3 independent experiments, and *P* < 0.05 indicates a significant difference.

## Results

### Effects of narciclasine on pulmonary injury

**Figure 1.**
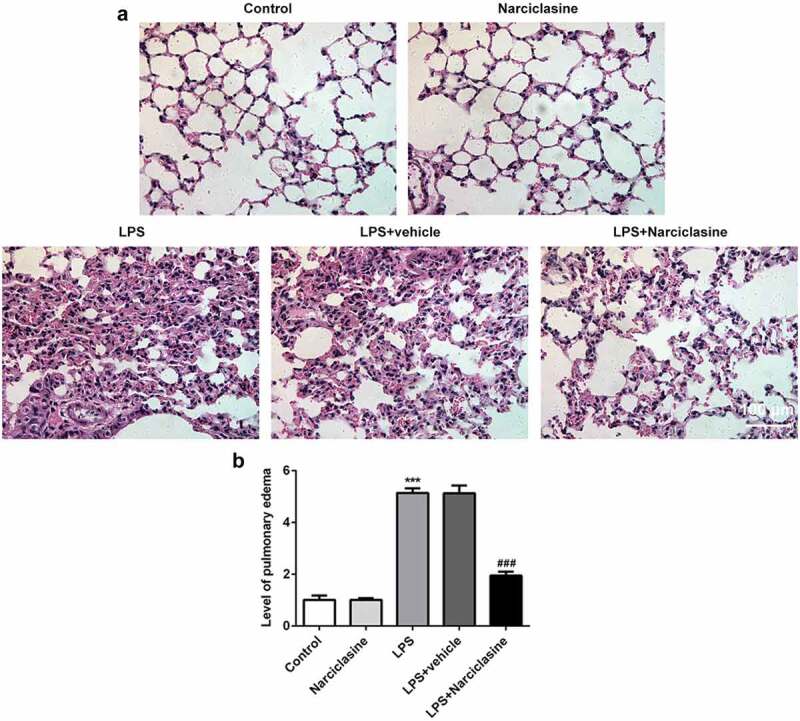
**Effects of narciclasine on pulmonary injury**. (a) Representative images of hematoxylin and eosin (H&E) staining on lung tissues in different groups (scale bars = 100 μm). (b) Pulmonary edema was determined by pulmonary edema score. Data are presented as the mean ± standard deviation (*n* = 5). ***P < 0.001 *vs*. Control; ^###^*P* < 0.001 *vs*. LPS.

HE staining and pulmonary edema score were employed to analyze the pathological morphology of lung tissue after pulmonary damage. There are no significant difference between control group and narciclasine. These results indicated that narciclasine have no toxicity in neonatal rats. Compared with control group, histological changes such as inflammation, hemorrhage, alveolar congestion, and alveolar wall edema were observed in the lung tissue of neonatal ALI rats (Figure 1(a)). Interestingly, narciclasine treatment ameliorated the pathological changes effectively. Furthermore, remarkably increased lung water content was observed in the LPS-induced ALI group compared with control group, but the pulmonary edema was alleviated by narciclasine treatment (Figure 1(b)). These results above suggested that narciclasine possessed protective effect on pulmonary injury induced by LPS stimulation.

### Effects of narciclasine on LPS-induced inflammatory response in lung tissue of ALI neonatal rats

**Figure 2.**
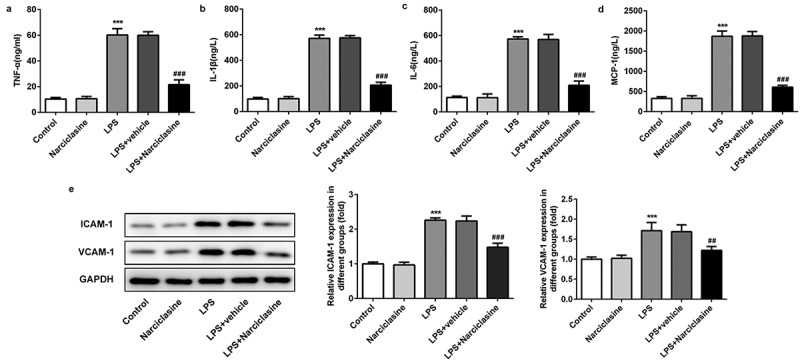
**Effects of narciclasine on LPS-induced inflammatory response in lung tissue of neonatal ALI rats**. (a–d) The amount of inflammatory cytokines including TNF-α, IL-1β, IL-6, and MCP-1 were determined through ELISA kits. (b) Expression levels of ICAM-1 and VCAM-1 were determined by Western blot. Data are presented as the mean ± standard deviation (*n* = 5). ***P < 0.001 *vs*. Control; ^##^*P* < 0.01, ^###^*P* < 0.001 *vs*. LPS.

ELISA kits were used to determine the levels of inﬂammatory factors such as tumor necrosis factor-α (TNF-α), Interleukin (IL-6), IL-1β, monocyte chemotactic protein-1 (MCP-1) in serum. Compared with the control group, the expression levels of TNF-α, IL-6, IL-1β and MCP-1 were significantly increased in serum of LPS-induced neonatal ALI rats. Interestingly, narciclasine treatment remarkably suppressed the inflammatory cytokines release (Figure 2(a–d)), indicating that narciclasine had anti-inflammation activities in LPS-induced ALI neonatal rats.

Cell adhesion molecules are crucial regulative factors in inflammatory response. The expressions of intercellular adhesion molecule-1 (ICAM-1) and vascular cell adhesion molecule-1 (VCAM-1) were determined by western blot. Compared with the control group, the expressions of ICAM-1and VCAM-1 were notably increased by LPS challenge, which were significantly reversed by narciclasine treatment. The results of western blot analysis further suggested that narciclasine exerted suppressive effect on LPS-induced excessive inflammation in lung tissue.

### Effects of narciclasine on oxidative stress state in lung tissues of neonatal ALI rats

**Figure 3.**
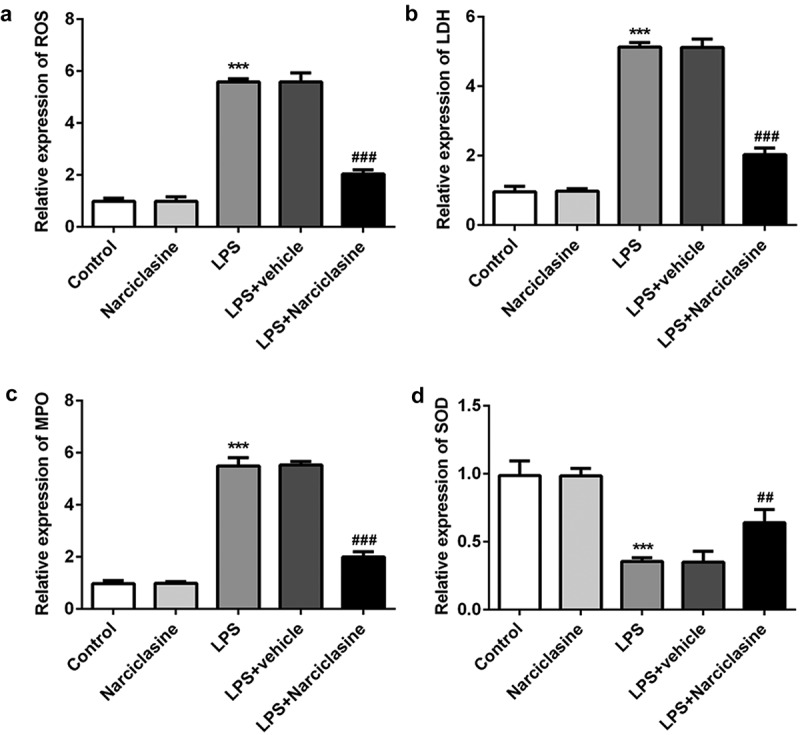
**Effects of narciclasine on oxidative stress statue in lung tissue of neonatal ALI rats**. (a) The amount of ROS was measures by commercial ROS assay kit. The activities of LDH (b), MPO (c), and SOD (d) were detected using specific commercial test kits. Data are presented as the mean ± standard deviation (*n* = 5). ***P < 0.001 *vs*. Control; ^##^*P* < 0.01, ^###^*P* < 0.001 *vs*. LPS.

The amount of reactive oxygen species (ROS) production and the antioxidant enzymes activities, including lactate dehydrogenase (LDH), superoxide dismutase (SOD), and myeloperoxidase (MPO) were detected using specific commercial assay kit, according to the manufacturer’s recommendations. Compared with the control group, the level of ROS production was increased significantly upon LPS stimulation. As except, narciclasine treatment decreased the amount of ROS production in lung tissue. As shown in Figure 3(b–d), the activity of SOD was decreased, and the activities of LDH and MPO were elevated in lung tissues of LPS-induced neonatal ALI rat model, which were all significantly reversed by narciclasine treatment. These results suggested narciclasine attenuated oxidative stress state in lung tissue of neonatal ALI rats

### Effects of narciclasine on cell apoptosis in lung tissue of neonatal ALI rats

**Figure 4.**
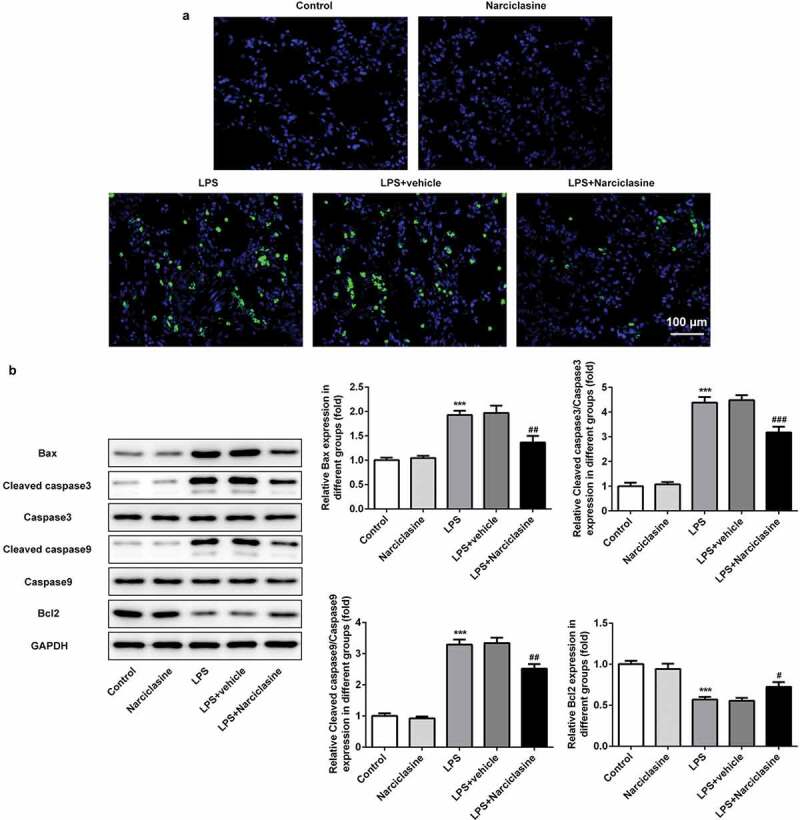
**Effects of narciclasine on cell apoptosis in lung tissue of neonatal ALI rats**. (a) Cell apoptosis in lung tissues was analyzed by TUNEL assay (scale bars = 100 μm). (b) Expression levels of proteins related to apoptosis, including Bcl2, Bax, Cleaved-caspase3, caspase3, Cleaved-caspase9, and caspase9, were determined by Western blot. Data are presented as the mean ± standard deviation (*n* = 5). ***P < 0.001 *vs*. Control; ^#^*P* < 0.05, ^##^*P* < 0.01, ^###^*P* < 0.001 *vs*. LPS.

TUNEL assay and western blot were performed to investigate the effect of narciclasine on cell apoptosis. As shown in Figure 4(a), The LPS-induced neonatal ALI rats exhibited higher FITC positivity in their lung tissues compared to control group, while narciclasine treatment notably reduced the cell apoptosis.

**Figure 5.**
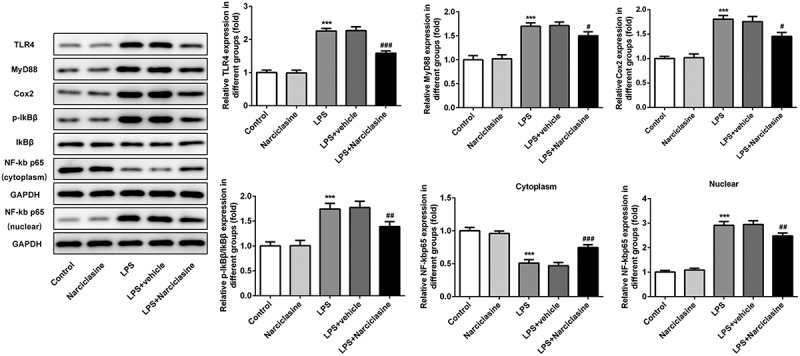
**Effects of narciclasine on TLR4/NF-κB/Cox2 signaling pathway**. Expression levels of proteins involved in TLR4/NF-κB/Cox2 signaling pathway, including TLR4, MyD88, Cox2, p- IκBβ, IκBβ, and NF-κB p65, were analyzed by Western blot. Data are presented as the mean ± standard deviation (*n* = 5). ***P < 0.001 *vs*. Control; ^#^*P* < 0.05, ^##^*P* < 0.01, ^###^*P* < 0.001 *vs*. LPS.

Meanwhile, the proteins related to cell apoptosis were determined by western blot to confirm the role of narciclasine in cell apoptosis. In comparison with control group, the expression levels of Bax, Cleaved caspase3 and Cleaved caspase9 in lung tissue of neonatal ALI rats were upregulated significantly. Narciclasine treatment significantly attenuated the expressions of Bax, Cleaved caspase3 and Cleaved caspase9. Caspase3 and caspase9 expressions have no significant modification. By contrast, the Bcl2 (anti-apoptotic protein) expression in lung tissue of neonatal ALI rats was decreased, while narciclasine treatment significantly increased the Bcl2 expression Figure 4(b). Above results suggested that narciclasine possessed protective effects on pulmonary injury by attenuating cell apoptosis rate.

### Effects of narciclasine on TLR4/NF-κB/Cox2 signaling pathway

To investigate the mechanism underlying the effect of narciclasine treatment, the proteins involved in toll-like receptor 4 (TLR4)/nuclear factor-κB (NF-κB)/Cyclooxygenase 2 (Cox2) signaling pathway were assessed by Western blotting. LPS challenge led to a significant increase in LPS-induced expressions of TLR, myeloid differentiation factor 88 (MyD88), Cox2 and phospho-inhibitor of κBβ (IκBβ), while narciclasine treatment led to a significant reduction in expressions of TLR, MyD88, Cox2 and p-IκBβ in lung tissue of neonatal ALI rats, with no significant difference in total IκBβ expression. In addition, LPS challenge induced a reduction in the expression of cytosolic NF-κB p65 but engendered an increase in the expression level of nuclear NF-κB p65, which were strikingly corrected by narciclasine treatment, as assessed by Western blot analysis Figure 5. Above proofs suggested administration of narciclasine significantly inhibited the activation the TLR4/NF-κB/Cox2 signaling pathway.

## Discussion

ALI is a lethal disease with high morbidity and mortality, which results from pneumonia, sepsis and other serious diseases [15]. Based on the anti-inflammatory properties of narciclasine, we presume that narciclasine may be an effective therapeutic agent for ALI therapy. In our study, lipopolysaccharide (LPS)-induced ALI animal model, the most well-established and popular model, was established to confirm the role of narciclasine in ALI and to investigate the molecular mechanisms involved in it. LPS is a specific biochemical component found in the outer membrane of Gram-negative bacteria [16]. Furthermore, LPS is demonstrated to be involved in excessive inﬂammatory cascade under various pathological conditions. It is well established that LPS-induced ALI is the most famous and popular animal models in literature, which has been performed extensively [17–19]. Hence, rats underwent LPS administration to establish neonatal ALI model in the present study. The ALI model was proved to be successful to produce consistent phenotypes with previous studies [13], such as inflammation, hemorrhage, alveolar congestion, and pulmonary edema. Interestingly, narciclasine treatment ameliorated the pathological changes effectively, indicating the protective effect of narciclasine in lung injury. Hence, further experiments were performed to investigate the potential mechanism responsible for the therapeutic effects of narciclasine on ALI.

In the present study, the levels of TNF-α, IL-6, IL-1β, and MCP-1, all of which are involved in pulmonary inflammation, were increased significantly in serum of LPS-induced neonatal ALI rats. The production of IL-6, a proinflammatory cytokines, is increased in bronchoalveolar lavage fluid (BALF) and lung tissue, when mice were exposed to particulate matter [20], which was consistent with our results. Moreover, Kai et al. demonstrated that saxagliptin alleviates the contents of inflammatory cytokine including TNF-α, IL-6, IL-1β, and MCP-1 in ALI treatment [21]. Recently, Kingsley et al reported that narciclasine protected against sepsis in neonatal rats via inhibition of calprotectin and pro-infammatory cytokines, including IL-2, IL-1α, and TNF-α [22]. In our study, narciclasine reduced the release of inflammatory cytokines, indicating the suppressive effect of narciclasine on uncontrolled inflammation-induced by LPS, which is consistent with previous study. In addition, cell adhesion molecules also play crucial regulative effect on inflammatory response. Previous studies have reported that the expressions of adhesion molecules were upregulated in injury site, which further provoked excessive inflammation [23]. Consistent with above study, LPS induced abnormal expressions of ICAM-1and VCAM-1 in lung tissue of ALI rat, which were corrected by narciclasine administration. These findings further confirm the anti-inflammation activity of narciclasine in ALI rats.

Oxidative stress plays a crucial role in the development of inﬂammatory disease, including ALI. Recent studies reported that saxagliptin alleviated oxidative stress via decreasing the levels of ROS, MPO and increasing the level of SOD in ALI animal model induced by LPS [21]. In the current study, narciclasine also suppressed the levels of ROS, LDH, and MPO, and enhanced the SOD level to protect against pulmonary injury. Furthermore, narciclasine decreased the expression of Bax and increased the expression of Bcl2, and dramatically reduced cell apoptosis in lung tissues of ALI rats. Consistent with our study, Jiang et al. reported that procyanidin B2 inhibited LPS-induced inflammation and apoptosis in human alveolar epithelial cells and lung fibroblasts by inactivating NF-κB and NLRP3 inflammasome signaling [24]. This evidence indicated that antiapoptosis may be another mechanism of lung protection from narciclasine.

Previous studies have shown that the inflammation induced by LPS was involved in activation of TLR/MyD88 pathway [25]. In the literature, narciclasine possesses strong anti-inflammation properties *in vitro* and *vivo* [10]. Hence, the TLR4/MyD88 pathway may mediate the role of narciclasine in ALI-induced by LPS. In our study, western blot results demonstrated that narciclasine inhibited the expression of TLR and MyD88, suggesting narciclasine inhibited TLR4 expression. Furthermore, TLR4 is well known as one of the major LPS signaling receptors and triggers oneset of NF-κB transcription [26]. When cytosolic NF-κB, as a complex consistent of p50, p65, and IκB, is activated by various stimulations, the generated phosphorylation and degradation of IκB will lead to NF-κB nuclear translocation, mediating inflammatory response progress. In addition, the increased production of ROS induced secretion of inflammatory cytokines and aggravate excessive inflammation via activation of NF-κB signaling pathway [27]. It is well known that inflammatory cytokines, including TNF-α, IL-6, and Cox2 are downstream regulators of NF-κB signaling. In the present study, the inhibitive effect of narciclasine on IκB-β phosphorylation indicated that narciclasine protects against lung injury via inhibiting NF-κB nuclear translocation. Moreover, narciclasine also suppressed the expressions of TLR4, MyD88, and Cox2 in lung tissue of ALI rats. Taken together, narciclasine attenuates inflammation, oxidative stress, and apoptosis via inactivation of TLR4/NF-κB/Cox2 signaling pathway.

## Conclusions

In summary, our study provides numerous proofs to confirm the lung protection of narciclasine. In the present study, narciclasine ameliorated the LPS-induced pathological changes in lung tissue of ALI rats. Furthermore, the treatment narciclasine suppressed excessive inflammatory response, oxidative stress and apoptosis via inactivation of TLR4/NF-κB/Cox2 signaling pathway. Most importantly, narciclasine may be considered as an effective and novel agent for the clinical therapeutic strategy of ALI treatment.

## Data Availability

The analyzed data sets generated during the present study are available from the corresponding author on reasonable request.

## Highlights

- Narciclasine possessed protective effect on pulmonary injury induced by LPS stimulation;
- Narciclasine inhibited LPS-induced inflammation, oxidative stress, and cell apoptosis in lung tissue of ALI neonatal rats;
- Narciclasine significantly inhibit the activation the TLR4/NF-κB/Cox2 signaling pathway.
